# Supplementary figures and images for: High-affinity P2Y2 and low-affinity P2X7 receptor interaction modulates ATP-mediated calcium signaling in murine osteoblasts
Source: PLoS Comput Biol. 2021 Jun 21;17(6):e1008872. doi: 10.1371/journal.pcbi.1008872 (PMC8248741; doi:10.1371/journal.pcbi.1008872)

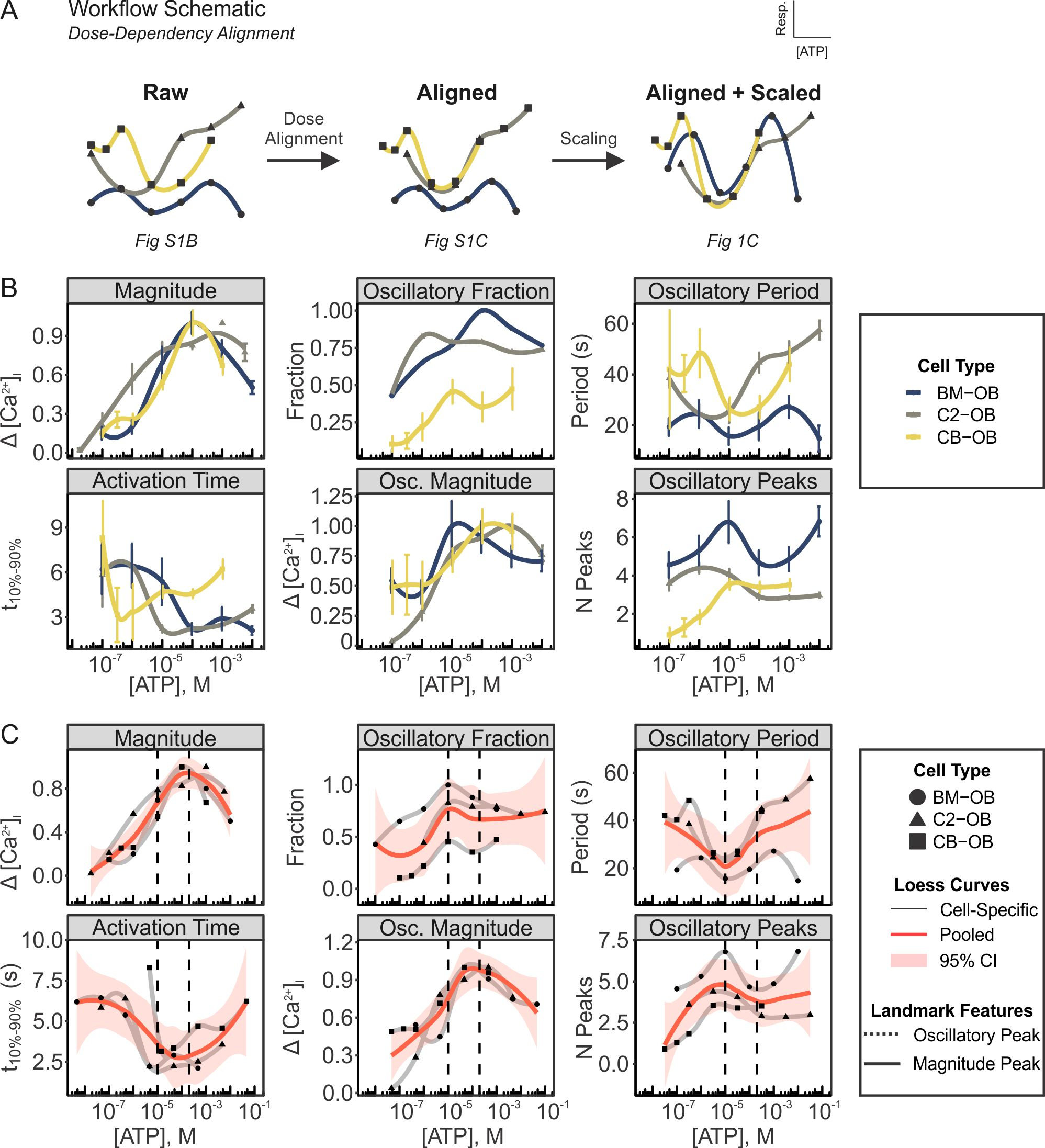

Supplement: S1 Fig — (A-C) Schematic illustrating processing of ATP-dose-dependent response curves (A). ATP dose-dependent responses from three independent murine cell lines (A, left panel; B) were aligned using a linear transformation to match peaks/troughs (A, middle panel; C) and responses were rescaled to [0,1] interval (A, right panel; Fig 1C). Curves: Loess curves; Markers: Response means (circle: BM-OB; triangle: C2-OB; square: CB-OB). (TIF) [file pcbi.1008872.s001.tif]
