# Supplementary material for: High-affinity P2Y2 and low-affinity P2X7 receptor interaction modulates ATP-mediated calcium signaling in murine osteoblasts
Source: PLoS Comput Biol. 2021 Jun 21;17(6):e1008872. doi: 10.1371/journal.pcbi.1008872 (PMC8248741; doi:10.1371/journal.pcbi.1008872)
Supplement: S1 Table — (PDF) [file pcbi.1008872.s002.pdf]

S1 Table. Calcium response parameters.

Parameter	Symbol	Units	Description
Activation time	$t_{10-90\%}$	s	Time for signal to increase from 10 to 90% of peak.
Magnitude	-	$\Delta[Ca^{2+}]_i$	Amplitude of response (peak – baseline).
Area under curve	AUC	$\Delta[Ca^{2+}]_i \cdot s$	Area under response curve.
Decay time	τ_{decay}	s	Decay constant of the exponential decay region of the deactivation phase of response.
Oscillatory fraction	-	-	Fraction of cells with multi-peaked (oscillatory response).
Oscillatory magnitude	E	$\Delta[Ca^{2+}]_i$	Mean magnitude of oscillatory peaks.
Oscillatory period	T	s	Mean periodicity of oscillatory peaks.
Oscillatory peaks	N_{osc}	-	Number of observed oscillatory peaks.
Response fraction	-	-	Fraction of cells with discernable calcium response.
Signal radius	ρ	µm	Average distance of secondary (2°) responders from primary (1°) stimulated cell.

Refer to [13] for schematic-based definitions.
