# Supplementary material for: High-affinity P2Y2 and low-affinity P2X7 receptor interaction modulates ATP-mediated calcium signaling in murine osteoblasts
Source: PLoS Comput Biol. 2021 Jun 21;17(6):e1008872. doi: 10.1371/journal.pcbi.1008872 (PMC8248741; doi:10.1371/journal.pcbi.1008872)
Supplement: S2 Table — (PDF) [file pcbi.1008872.s003.pdf]

S2 Table. Primer and PAM sequences		
**Target**	**Description**	**Sequence (5' – 3')**
P2ry2	CRISPR/Cas9; PAM sequence	Strand A: TTGGTTTATTACTACGCCCG<br>Strand B: CGGGCGTAGTAATAAACCAA
P2rx7	CRISPR/Cas9; PAM sequence	Strand A: CTGACCGGCGTTGTAAAAAG<br>Strand B: GAGCACTGTGCACCGCGCCT
P2ry1	qRT-PCR; primers	Fwd: AGCAGAATGGAGACACGAGTTTG<br>Rv: GGGATGTCTTGTGACCATGTTACA
P2ry2	qRT-PCR; primers	Fwd: GAAGAACTGGAGCAGGCGCT<br>Rv: CCATTGCCCTGGACCTGATC
P2ry4	qRT-PCR; primers	Fwd: CTGCAAGTTCGTCCGCTTTC<br>Rv: GTATTGCCCGCAGTGGATG
P2ry6	qRT-PCR; primers	Fwd: TGAAAACAACGAGGAACACCAA<br>Rv: CAGCCTTTCCTATGCTCGGA
P2ry12	qRT-PCR; primers	Fwd: CCGGAGACACTCATATCCTTC<br>Rv: GCCCAGATGACAACAGAAAG
P2ry13	qRT-PCR; primers	Fwd: CAGCTGAGTCTCTTCCAAAACAAA<br>Rv: TGCATCCCAGTGGTGTTGAT
P2ry14	qRT-PCR; primers	Fwd: CCACCACAGACCCTCCAAAC<br>Rv: CAACACGGGAATGATCTGCTTT
P2rx1	qRT-PCR; primers	Fwd: CAGAAAGGAAAGCCCAAGGTATT<br>Rv: CACGTCTTCACAGTGCCATTG
P2rx2	qRT-PCR; primers	Fwd: GCTGCTCATTCTGCTTTACTTCG<br>Rv: TCCCACACTTTGTGTTCCGA
P2rx3	qRT-PCR; primers	Fwd: AAGGCTTCGGACGCTATGC<br>Rv: GATGACAAAGACAGAAGTGCCCT
P2rx4	qRT-PCR; primers	Fwd: AGACGGACCAGTGATGCCTAAC<br>Rv: TGGAGTGGAGACCGAGTGAGA
P2rx5	qRT-PCR; primers	Fwd: GATGTGGCAGACTTTGTCATTCC<br>Rv: CCTTCACGCTCAGCACAGATG
P2rx6	qRT-PCR; primers	Fwd: ACGTGTTCTTCCTGGTAACCAACT<br>Rv: TGGACATCTGCCCTGGACTT
P2rx7	qRT-PCR; primers	Fwd: ACAATGTGGAAAAGCGGACG<br>Rv: TCAATGCACACAGTGGCCA
Gapdh	qRT-PCR; primers	Fwd: CAAGTATGATGACATCAAGAAGGTGG<br>Rv: GGAAGA-GTGGGAGTTGCTGTTG
