# Supplementary material for: High-affinity P2Y2 and low-affinity P2X7 receptor interaction modulates ATP-mediated calcium signaling in murine osteoblasts
Source: PLoS Comput Biol. 2021 Jun 21;17(6):e1008872. doi: 10.1371/journal.pcbi.1008872 (PMC8248741; doi:10.1371/journal.pcbi.1008872)
Supplement: S3 Table — (PDF) [file pcbi.1008872.s004.pdf]

S3 Table. PCR cycling conditions

Stage	Description	Cycles (n)
**qRT-PCR (cDNA template)**		
**Phase 1**	Denaturation: 94 °C, 180 s	1
**Phase 2**	Denaturation 94 °C, 30 s<br>Annealing: 60 °C, 45 s<br>Elongation: 72 C, 60 s	40
**Phase 3**	Elongation: 72 °C, 600 s<br>Hold: 4 °C	1
