# Supplementary material for: High-affinity P2Y2 and low-affinity P2X7 receptor interaction modulates ATP-mediated calcium signaling in murine osteoblasts
Source: PLoS Comput Biol. 2021 Jun 21;17(6):e1008872. doi: 10.1371/journal.pcbi.1008872 (PMC8248741; doi:10.1371/journal.pcbi.1008872)
Supplement: S1 Text — (PDF) [file pcbi.1008872.s005.pdf]

S1 Text. Solutions and Reagents

***Solutions*.** Phosphate-buffered saline (PBS; 140 mM NaCl, 3 mM KCl, 10 mM Na_2HPO_4, 2 mM KH_2PO_4, pH 7.4), autoclaved; Phosphate buffered saline with Tween 20 (PBST; PBS + 1% Tween 20); Physiological solution (PS; 130 mM NaCl, 5 mM KCl, 1 mM $MgCl_2$, 1 mM $CaCl_2$, 10 mM glucose, 20 mM HEPES, pH 7.6), sterilized by 0.2 µm filtration; RIPA lysis buffer (50 mM Tris, pH 7.4, 150 mM NaCl, 1% Nonidet P-40, 1 mM EDTA, 1 mg/mL aprotinin, 2 mg/mL leupeptin, 0.1 mM phenylmethylsulfonyl fluoride, 20 mM NaF, 0.5 mM Na3VO4); TBST buffer (10 mM Tris-HCL, pH 7.5, 150 mM NaCl, 1% Tween 20).

Reagents. High-capacity cDNA reverse transcription kit (Cat. 4368814); *Power* SYBR Green Master Mix (Cat. 4368702) from Applied Biosystems. Nitrocellulose membrane, 0.45 µm (Cat. 162-0115) from Bio-Rad. Opti-MEM (Cat. 31985062) from Gibco. Fura2-AM (Cat. F1221); Lipofectamine 3000 transfection reagent (Cat. L3000001); Quant-iT protein assay kit (Cat. Q33210) from Invitrogen. Puromycin (Cat. ant-pr-1) from InvivoGen. 35 mm glass-bottom dishes (Cat. P35G-1.5-14-C); 48-well glass-bottom plates (Cat. P48G-1.5-6-F) from MatTek Corporation. RNeasy Mini Kit (Cat. 74104) from Qiagen. Adenosine 5'-triphosphate (ATP; Cat. A9187); Venor GeM Mycoplasma PCR-based detection kit (Cat. MP0025) from Sigma-Aldrich. Pierce ECL western blotting substrate (Cat. 32106) from Thermo Scientific. Dulbecco's modified eagle medium (DMEM; Cat. 319-020 CL); Fetal bovine serum (FBS; Cat. 080152); Penicillin streptomycin (Cat. 450-201-EL); Sodium pyruvate (Cat. 600-110-UL) from Wisent Bio Products.
